# Evolving Nutritional Strategies in the Presence of Competition: A Geometric Agent-Based Model

**DOI:** 10.1371/journal.pcbi.1004111

**Published:** 2015-03-27

**Authors:** Alistair M. Senior, Michael A. Charleston, Mathieu Lihoreau, Camille Buhl, David Raubenheimer, Stephen J. Simpson

**Affiliations:** 1 Charles Perkins Centre, The University of Sydney, Sydney, New South Wales, Australia; 2 School of Biological Sciences, The University of Sydney, Sydney, New South Wales, Australia; 3 School of Information Technologies, The University of Sydney, Sydney, New South Wales, Australia; 4 Centre National de la Recherche Scientifique (CNRS), Centre de Recherches sur la Cognition Animale, Toulouse, France; 5 School of Agriculture, Food and Wine, The University of Adelaide, Adelaide South Australia, Australia; 6 Faculty of Veterinary Science, The University of Sydney, Sydney, New South Wales, Australia; 7 Université Paul Sabatier (UPS), Centre de Recherches sur la Cognition Animale, Toulouse, France; Instituto Superior Técnico, Univesidade de Lisboa, PORTUGAL

## Abstract

Access to nutrients is a key factor governing development, reproduction and ultimately fitness. Within social groups, contest-competition can fundamentally affect nutrient access, potentially leading to reproductive asymmetry among individuals. Previously, agent-based models have been combined with the Geometric Framework of nutrition to provide insight into how nutrition and social interactions affect one another. Here, we expand this modelling approach by incorporating evolutionary algorithms to explore how contest-competition over nutrient acquisition might affect the evolution of animal nutritional strategies. Specifically, we model tolerance of nutrient excesses and deficits when ingesting nutritionally imbalanced foods, which we term ‘nutritional latitude’; a higher degree of nutritional latitude constitutes a higher tolerance of nutritional excess and deficit. Our results indicate that a transition between two alternative strategies occurs at moderate to high levels of competition. When competition is low, individuals display a low level of nutritional latitude and regularly switch foods in search of an optimum. When food is scarce and contest-competition is intense, high nutritional latitude appears optimal, and individuals continue to consume an imbalanced food for longer periods before attempting to switch to an alternative. However, the relative balance of nutrients within available foods also strongly influences at what levels of competition, if any, transitions between these two strategies occur. Our models imply that competition combined with reproductive skew in social groups can play a role in the evolution of diet breadth. We discuss how the integration of agent-based, nutritional and evolutionary modelling may be applied in future studies to further understand the evolution of nutritional strategies across social and ecological contexts.

## Author Summary

Getting enough nutrients and at the right balance is among the primary challenges that an animal has to overcome. Animals that live in groups have the added complexity of competition among individuals over foods. We used an evolutionary simulation to explore how the intensity of such competition interacts with the composition of available foods to influence the strategies that an animal should use to meet its nutritional requirements. We found that two general strategies emerged. When competition was weak, animals that only locate and consume foods with an ideal balance of nutrients were favoured. However, when competition was strong, a strategy with which animals meet their nutritional requirements by eating large amounts of nutritionally imbalanced, but complementary, foods was optimal. These results implicate a role for competition for foods between animals within social groups in shaping dietary breadth. Evolutionary simulations such as those described here are important for understanding how different species evolve to meet their nutritional requirements in a range of ecological circumstances.

## Introduction

Access to nutrients is one of the most influential factors affecting reproductive development and ultimately fitness (e.g. [1–4]). A range of factors can influence nutrient access, but for many organisms interactions with conspecifics are pivotal. Group living animals in particular face a complex trade-off between access to foods that provide them with a balanced diet, social interactions that enhance fitness via benefits of group cohesion, and competition [5]. Contest-competition, for example, where individuals directly engage one another for access to nutrients, is a source of inter-individual variance that can lead to clear dominance hierarchies [6,7]. In the extreme, contests may even lead to a reproductive division of labour, with only those individuals at the top of the hierarchy being able to access enough nutrients, and at the right balance, to reproduce [8–10].

The effects that competition over food access can have on inter-individual variation in reproduction are well known in arthropods. For example, colonies of social spiders (e.g. *Stegodyphus sp*.) tend to be characterised by body size asymmetries and reproductive skews [11–15]. As a result of contest-competition over food access, only larger females are able to attain enough of the right nutrients to reproduce [8,13]. It has even been proposed that the reproductive asymmetries that arise from competition over nutrients may constitute a reproductive division of labour, in which non-reproductive spiders provide alloparental care ([8,16] c.f. [14,17]). In the burying beetle, *Nicrophorus vespilloides*, females compete for access to carcasses, which in turn leads to a dominance hierarchy where reproduction is skewed in favour of the dominant female [18]. Experimental data indicate that access to appropriate nutrition is the main factor determining reproductive output and also impacts performance in dominance interactions [18,19]. Although there is no such direct evidence in cooperative breeding vertebrates, correlative studies in mongooses (*Mungos mungo*) and meerkats (*Suricata suricatta*) show that subordinate females breed more frequently in periods of food abundance [20,21]. These results highlight the importance of contest competition as a potentially major ecological factor shaping the evolution of nutritional and social traits in animal groups.

**Fig 1:**
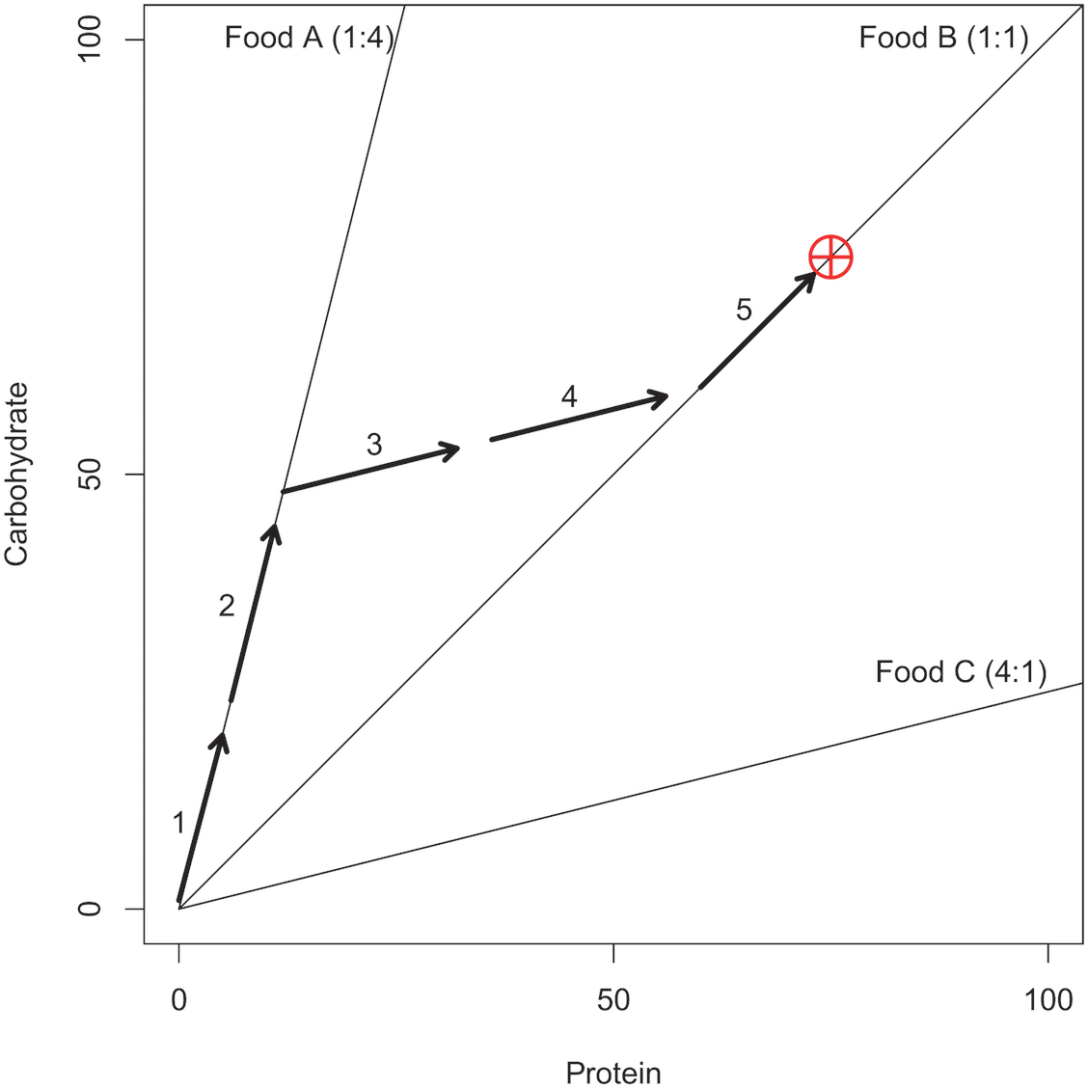
An Example of the Geometric Framework (GF). A visualisation of the GF [23] used to track the nutritional state of a hypothetical individual. The graph area depicts the nutrient space available with two macronutrients; protein (P) is represented on the *x*-axis and carbohydrate (C) on the *y*-axis. The requirements of the individual with regards the two macronutrients are depicted by the Intake Target (IT; the red crosshair). In this instance the requirements for P and C are equal (75:75). An individual’s nutritional state is its (*x*, *y*) position in the nutrient space. The only way for an individual to move through the nutrient space to the IT is to eat foods. Three foods with differing P:C ratios are depicted by three food rails (solid black lines); Food A is high in C but low in P (P:C, 1:4); Food B is balanced (1:1); Food C is high in P but low in C (4:1). As an individual eats, its nutritional state moves through the nutrient space (depicted by the sequence of arrows) in parallel to the food rail for the food it is eating. Here, the individual has reached the IT by first eating Food A for steps one and two, then Food C for steps three and four, and finally on Food B for the step five. The individual could also have taken a more direct route to the IT by eating only Food B. This food is nutritionally balanced in regard to the individual’s IT. Alternatively, the individual may have eaten equal amounts of macronutrient from Foods C and A. These two foods are individually imbalanced but collectively complementary.

In recent years, a new understanding of interactions between an organism’s nutritional requirements and its environment has been gained using the state-space models of the Geometric Framework (GF) [22–24]. In the GF, nutrients are represented by a Cartesian coordinate system, which is referred to as the nutrient space [23]. In the simple case of a nutrient plane, the GF represents two food components (e.g., the macronutrients protein and carbohydrate) on *x* and *y* axes (Fig. 1). The optimal amount and blend of nutrients that the animal requires over a specified period in its life are represented by a coordinate or region within the nutrient space called the Intake Target (IT; [23]). Foods are represented by ‘food rails’, which are radials through the nutrient space with a slope that reflects the ratio of macronutrients present within the food (Fig. 1). As an individual eats, its nutritional state (*x*, *y* coordinate) moves through the nutrient space in parallel with the rail of the food it consumes. A high quality food may be considered one with a food rail that will guide an individual’s nutritional state to the IT from its current state (Fig. 1); i.e., one that is nutritionally balanced. When confined to nutritionally imbalanced foods, the animal needs to resolve the trade-off between over-ingesting some nutrients and under-ingesting others. The strategy that it adopts in this situation, known as the ‘rule of compromise’, is expected to vary within and between species depending on the relative costs of ingesting excesses and deficits of the different nutrients [23,25].

Recently, the GF has been combined with Agent-Based Models (ABMs; simulations representing each individual explicitly, sometimes named individual-based models in ecological fields [26]) to successfully demonstrate how social interactions and nutritional strategies affect one another [5]. With regard to the influence of competition on the emergence of reproductive asymmetries, Lihoreau et al. [5] link classic models of contest competition (outlined by Bonabeau et al. [27]) with the GF in an ABM. In that model, access to each food rail is limited, and individuals must displace competitors via dominance interactions before feeding. Performance in dominance interactions is a function of the individual’s fitness, which in turn is negatively correlated with the distance between an individual’s nutritional state and the IT. Reproductive asymmetry arises as individuals who ‘get lucky’ and are able to feed on high quality foods early experience a ‘winner effect’ (see [28]). That is, a loop of positive feedback ensues whereby better-nourished individuals continue to perform well in dominance interactions and monopolise high quality foods. Ultimately, only certain individuals attain enough nutrients at the right balance to breed, a model outcome that is consistent with observations of reproductive skew in some social animals (e.g. spiders [8,13] and burying beetles [18,19]). Interestingly, this model also clearly demonstrates how early stochasticity in nutrient access can lead to the emergence of a self-organised social structure from an initially homogeneous group [5,29].

The aforementioned mechanistic model, however, does not consider the optimal nutritional strategy that individuals should adopt. When feeding on a poor quality food, an individual may choose to stop eating and seek an alternative. However, the individual risks incurring costs; e.g., the time spent attempting, but ultimately failing, to gain access to alternative better foods. Under some circumstances it is thus conceivable that an individual could get its nutritional state closer to the IT by consuming a poor quality food, rather than by frequently searching for better balanced alternatives. Ultimately, the optimal strategy for leaving a suboptimal food may be dependent on the level of competition and the kinds of food in the environment.

The incorporation of evolutionary and genetic components into GF based ABMs has been identified as a promising method with which to understand how ecological factors interact with nutritional strategies [5,23,30]. Here, we present the first such model, which we used to explore how intra-specific competition might affect the evolution of animals’ nutritional strategies. In the model by Lihoreau et al. [5], an individual’s nutritional strategy was governed by the fixed global parameter *K*, which we refer to here as ‘nutritional latitude’. When eating a food that will not guide its nutritional state to the IT an individual has some probability of leaving, which is both a function of the balance of nutrients in the food being consumed, and *K*. Here, a high *K* means an individual is likely to consume the same imbalanced food until reaching a point of nutritional compromise (at which point it then seeks an alternative). In contrast, a low *K* corresponds to a low probability that an individual will continue feeding on a food rail that will not guide its nutritional state directly to the IT. Individuals with extremely high or low values of *K* may, thus, be thought of as nutrient generalists or specialists, respectively (*sensu* Raubenheimer and Simpson [31]; we note that *K* as we model it here is equivalent to 1—*K* in Lihoreau et al. [5]).

In this study, we couple nutritional latitude with an evolutionary algorithm, whereby an individual’s *K* is governed by an individual-level, heritable and mutable value. Each generation consists of 150 individuals that must attain a certain level of fitness (i.e., nutritional state) within a fixed number of model iterations for it to be considered fit enough to breed. Fitness-proportionate selection then operates among those individuals fit enough to breed, with proximity to the IT (optimal point of nutrient intake in the nutrient space) determining this fitness. We allowed *K* to evolve over 1000 generations under varying levels of competition and in differing nutritional environments (i.e., different abundance and nutritional compositions of food). In doing so, we aimed to explore the effects of contest competition and the number and composition of foods in the nutritional environment on the evolution of individual nutritional strategies.

## Results

### Experiment 1: Contest Competition in 2- and 3-Food Environments

We began by exploring the effect of intensity of competition on the evolution of nutritional latitude in 2- and 3-food environments. We performed 30 model runs under varying intensities of competition (*c*, which is bounded at 0 and 1; all parameters are outlined in Table 1, and their mode of action is described in Models). From each model run we recorded the population mean nutritional latitude (*K*, also bounded at 0 and 1) after 1000 generations.

**Table 1:** Model parameters and nomenclature.

Name	Notation	Description	Value
Global Parameters and Variables			
Intake Target	IT	The intake target is an (*x*, *y*) coordinate in the nutrient space that depicts the ideal amount and blend of nutrients an individual.	(500, 500)
Food Value	*V*	The amount of carbohydrate in a food for 1 unit of protein. Note that there is a separate *V* for each food in the environment.	Combinations of 0.0625, 0.5, 1, 2 and 16 were used.
Food Abundance	*a*	A variable that governs the number of individuals that can feed at any one time and thus the level of competition, via Equations 1 and 3 (note that in Lihoreau et al. [5], this parameter is termed *c*).	Values of 0.1 through *N*_*food*_ were assessed.
Number of Foods	*N*_*food*_	The number of different food sources that are available at any one time.	2 or 3.
Competition	*c*	The intensity of competition present in the model, which was manipulated by varying food abundance, *a*.	Equation 3.
Maximum Consumption	*φ*	The maximum amount of food that an individual can eat in one time step.	2 (see 'Initialisation')
Feeding Individuals	*N*_*ind*_	The number of individuals in the environment.	150
Dominance Constant	*η*	A constant denoting how difference in fitness affects the probability of the outcome of a dominance interaction.	10, 20 and 25; see [5].
Fitness Constant	*μ*	A constant denoting how distance from the IT affects fitness.	2; see [5].
Individual Parameters and Variables			
Nutritional State	*NS*	An individuals (*x*, *y*) coordinate within the environment.	Variable (*x*, *y*)
Fitness	*F*	The fitness of an individual, which is maximised when the individual reaches the IT.	Equation 6.
Appetite	*A*	The amount of food an individual would eat to get as close as possible to the IT on the food being consumed; i.e. how far would an individual move through the space to maximise its fitness on a given food.	Equation 5.
Nutritional Latitude	*K*	An individual, heritable and mutable variable that determines how likely an individual is to leave an inadequate food source—*P*_*leave*_.	Heritable.
Probability of Dominance	*Q*_*ij*_	The probability of the *i*^*th*^ individual displacing the *j*^*th*^ in a dominance interaction.	Equation 2.
Ideal Vector	*V*_*T*_	The hypothetical vector along which an individual would travel to reach the IT on an ‘ideal’ food.	Variable (see ‘Eat‘)
Angle of the Food Rail	*a*_*f*_	The angle of the food rail on which the individual is feeding.	Variable (see ‘Eat‘)
Angle of the 'Ideal' Food Rail	*α*_*ideal*_	The angle of the ideal food rail that would connect the individual's nutritional state with the IT.	Variable (see ‘Eat‘)
Distance to IT	*D*_*N*_	The Euclidean distance between the individual's nutritional state and the IT. For the calculation of fitness *F*, this is treated as the proportion of the total distance an individual must cover over the period of the simulation to reach the IT (i.e. equivalent to [5]).	Variable
Probability of Leaving Food	*P*_*leave*_	The probability that an individual will spontaneously leave an inadequate food source, which is governed by *K* and the quality of food.	Equation 7.

Parameters with our model, their notation, description and modelled values thereof.

**Fig 2:**
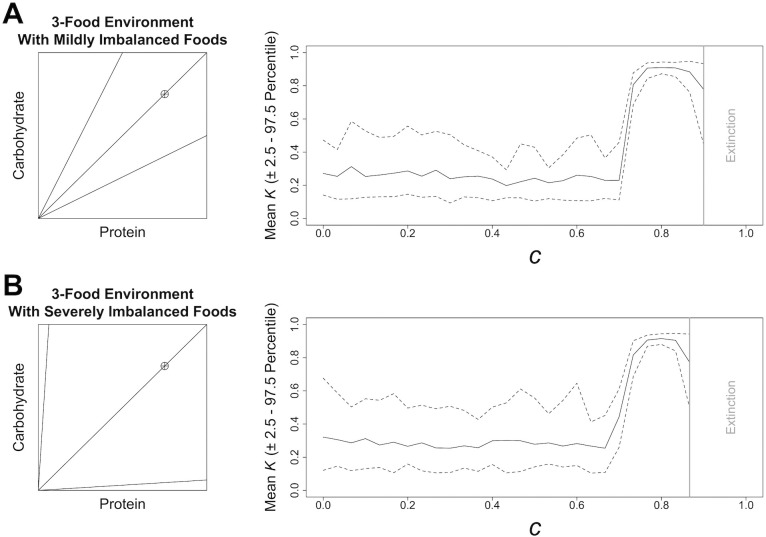
Model Results in Different 3-Food Environments. The effects of increasing competition, *c* (Equation 3), on the mean level of nutritional latitude, *K*, (± the 2.5^th^ and 97.5^th^ percentile; dashed line) that is stable under differing nutritional environments containing 3 foods. Data are based on 30 model runs. Data from levels of competition above which the population could not consistently survive (i.e., extinction, given by a bold grey line) have been removed. A geometric visualisation of each environment is given; lines and a crosshair depict food rails and the intake target, respectively.

We first looked at the effects of competition in environments containing one nutritionally balanced food, and two imbalanced but complementary foods (i.e., those which between them subtend a region of the nutrient space containing the IT). For the latter two complementary foods we varied the extent of their nutritional imbalance (Fig. 2). In these environments when *c* = 0, *K* was stable at a range of values (Fig. 2). The high variance in stable values of *K* suggests that no one level of nutritional latitude is optimal where competition is weak, but most low levels are equally fit. In the face of increasing *c*, *K* was relatively stable up to a point. With mildly imbalanced foods at *c* = 0.7, and with extremely imbalanced foods at *c* = 0.67, *K* increased sharply to above 0.91 (Fig. 2). In both 3-food environments, increases in *K* were accompanied by declines in the variance of evolved *K* (Fig. 2). For example, in the environment with mildly imbalanced foods the 2.5^th^ and 97.5^th^ percentiles of *K* were 0.14 and 0.47, respectively with *c* = 0, but were 0.69 and 0.88 when *c* = 0.73 (Fig. 2A). Thus, selection for a high level of nutritional latitude is strong at moderate to high *c*, with a further sharp decrease at extremely high levels of *c* (i.e., > 0.85), largely being driven by a change in the lower 2.5^th^ percentile of *K* (Fig. 2). At very high *c* the population could not support itself as no individuals could fulfil the fitness requirements to be considered in breeding condition by the end of the simulation (Fig. 2).

**Fig 3:**
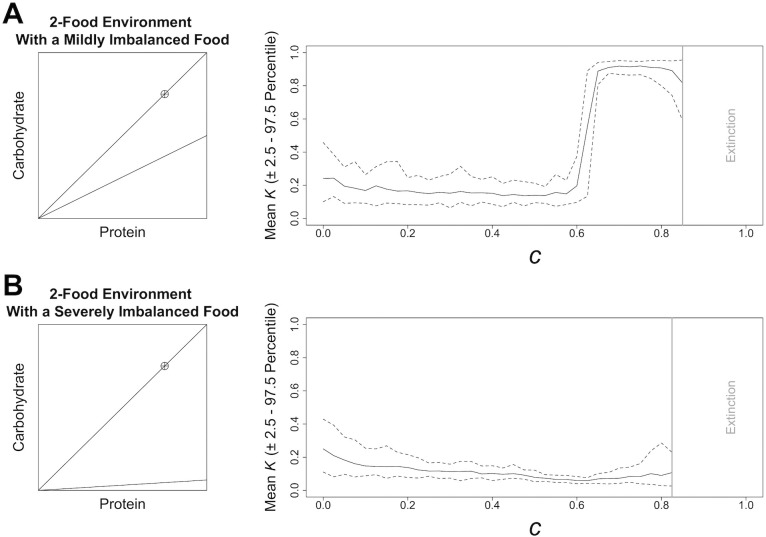
Model Results in Different 2-Food Environments. The effects of increasing competition, *c* (Equation 3), on the mean level of nutritional latitude, *K*, (and the 2.5^th^ and 97.5^th^ percentile; dashed line) that is stable under differing nutritional environments containing 2 foods. Data are based on 30 model runs. Data from levels of competition, above which the population could not consistently survive (i.e., extinction, given by a bold grey line), have been removed. A geometric visualisation of each environment is given; see Fig. 2 legend for details.

We next considered competition in a 2-food environment, containing one balanced and one imbalanced food, the latter of which varied in the degree of nutritional imbalance (Fig. 3). With a mildly imbalanced food, absent or weak competition selected for a lower *K* (and lower variance; Fig. 3A) than in 3-food environments (Fig. 2). Thus, selection for low nutritional latitude was stronger in this 2-food environment than in the 3-food environments. That being said, in the 2-food environment with a mildly imbalanced food and low *c*, *K* was stable, before transitioning to high *K* under moderate to high *c* (Fig. 3A), as was the case in 3-food environments (Fig. 2). In the 2-food environment that contained a balanced and a severely imbalanced food, nutritional latitude showed a quite different profile from that previously observed. Increasing *c* in this environment selected for low nutritional latitude (and very low variance in *K*), reaching a minimum value of *K* = 0.06 at *c* = 0.625 (Fig. 3B).

These results clearly indicate the importance of access to a complementary food to correct the nutritional state associated with consuming large amounts of a severely nutritionally imbalanced food (see S1 File for additional discussion). As part of experiment 1, we also looked at environments containing two nutritionally imbalanced but complementary foods. In these environments, the response of *K* to increasing *c* resembled that in 3-food environments (see S1 File).

### Experiment 2: Underlying Mechanisms

#### Proximate mechanism

To understand the mechanism underlying the effects of competition on nutritional latitude observed in Experiment 1, we allowed the model to run for a single generation (500 iterations), with 150 individuals, half of which expressed high nutritional latitude (*K* = 0.85) and half low nutritional latitude (*K* = 0.25). For this experiment we used a 3-food environment containing one balanced and two moderately imbalanced, but nutritionally complementary foods. The performance of the two strategies was assessed at a moderate level of competition (*c* = 0.683), where low *K* appears optimal and at a higher level of competition (*c* = 0.767), which selected for high *K*. We performed 30 replicates of each parameter set and recorded five variables. First, we recorded for each individual the mean of the angular difference between the food rail for the food consumed at each time step and the ideal food rail that would guide them to the IT (*β*, see Models, Equation 5). A smaller *β* indicates an animal that has taken a more direct route to the IT; i.e. a mean *β* = 0 would be an animal that only ate foods with the target balance of nutrients (e.g. Food B, Fig. 1). Second, we recorded the number of iterations that each individual spent displaced from food by the end of simulation. This is a measure of the rate at which an animal eats and thus accrues fitness. Third, we recorded the fitness (*F*) of each individual (see Models, Equation 6). Fourth, we recorded the cumulative number of contests that an individual initiated as the simulation progressed. Finally, we recorded the proportion of all contests that an individual won relative to the number of contests they were involved in (i.e. win rate; referred to as dominance index in [5]).

Where competition was moderate (where *c* = 0.683), individuals with low *K* had the lowest mean *β*, indicating that they ate more foods with an ideal nutritional balance (low *K*; *β* = 30.97; high *K*; *β* = 36.48). However, low *K* also resulted in a greater mean number of iterations for which individuals were displaced from food by the end of the simulation (low *K*; 30.93 iterations; high *K*; 22.71 iterations). Where competition was more intense (*c* = 0.767), low *K* still resulted in a lower *β* (low *K*; *β* = 11.39; high *K*; *β* = 17.38), but at the cost of a much greater mean number of iterations displaced from food at the end of simulation (low *K*; 174.9 iterations; high *K*; 124.5 iterations). Individuals with a lower *K* value thus sacrificed the short-term advantage of rapid movement through the nutrient space (by spending more time displaced from food), for the long-term benefit of attaining a nutritional state closer to the IT (by maintaining a low mean *β*).

**Fig 4:**
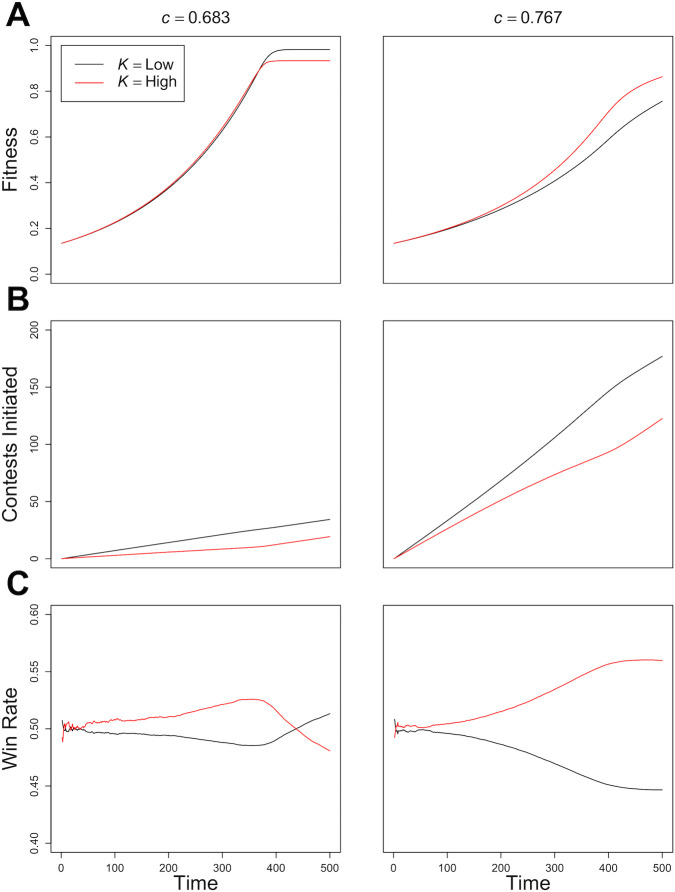
The Effects of *K* on Variables Within a Single Generation Over Time. The mean A) fitness, B) number of contests and C) win rate of individuals with *K* = 0.25 (black) and *K* = 0.85 (red) over time within one generation, when competition, *c* (Equation 3) is 0.683 (left panels) and 0.767 (right panels). Each panel is based on the results of 30 independent model runs, each with 75 individuals with each *K* value.

The effects of these different *K* strategies on fitness under different levels of competition are shown in Fig. 4A. Where *c* = 0.683, differences in the rate that individuals accrue *F* are small and ultimately by the end of the simulation individuals with low *K* (as a result of maintaining low *β*) are nearer the IT and have the highest fitness (Fig. 4A). In contrast, where *c* = 0.767 differences in the rate of fitness gain between *K* strategies are high; high *K* being associated with most rapid increases in *F*, a difference that becomes more pronounced as the simulation progresses (Fig. 4A). The increasing gulf in fitness is explained by the fact that individuals with a low *K* engage in more contests in an attempt to maintain a low *β* (i.e. obtain balanced foods; Fig. 4B). Where competition is weaker, eventually individuals with low *K* begin to win more contests because the differences in the rate of fitness gains are small and the long-term fitness benefits of maintaining low *β* begin to pay off (Fig. 4C). In contrast, where *c* = 0.767 low *K* individuals still engage in a high number of contests (Fig. 4B), but have a very low relative fitness (a result of having spent a large number of iterations displaced from food), and as a result have a low win rate (Fig. 4C). In turn, where competition is intense, the poor win rate, large number of contests and relatively poor fitness associated with low *K* feedback negatively on one another.

#### Evolutionary dynamics

Exploratory analysis suggested that frequency dependent effects might underlie the rapid shift from low to high nutritional latitude with increasing competition (Figs. 2 and 3). To further explore this idea we repeated the single generation experiment, but co-varied the proportion of the population adopting low and high *K* strategies (*K* = 0.25 and *K* = 0.85) and the level of competition (*c*). At the end of each replicate model run, we recorded the mean fitness of individuals with each *K*.

**Fig 5:**
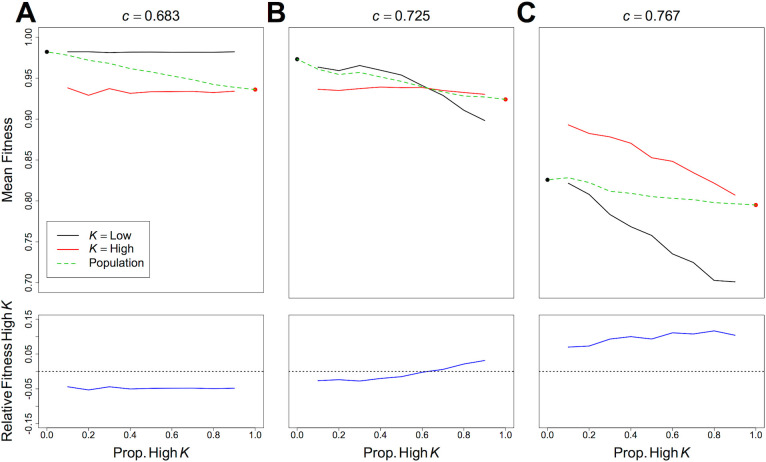
The Effects of the Proportion of the population With High *K* on Fitness at the End of a Single Generation. The effects of the proportion of the population with high *K* (*K* = 0.85) on the fitness of mean fitness of low *K* individuals (*K* = 0.25; black lines), high *K* individuals (red lines), the whole population (dashed green lines), and the relative fitness of high K individuals (fitness of high *K*—fitness of low *K*; blue line lower panels) at the end of a generation, under A) *c* (Equation 3) = 0.683, B) *c* = 0.725 and C) *c* = 0.767.

The effects of the frequency of individuals with high nutritional latitude (*K*) on the fitness at *c* = 0.683, 0.725 and 0.767 (values over which a transition between optimal strategies occurs) are given in Fig. 5. Where competition was weaker, low *K* individuals had the highest fitness, regardless of the proportion of the population with high *K*. In this situation, high *K* will not be selected for, clearly being the least fit strategy, regardless of its prevalence. Where competition is more intense (*c* = 0.725), increasing the frequency high *K* individuals decreases the mean fitness of low *K* individuals to a large extent (Fig. 5B). As a result, when at a high frequency (i.e. > 60% of the population) high *K* becomes the fittest strategy relative to low *K*. It is worth noting that when *c* = 0.725, high *K* would only be selected for once the prevalence of strategy reached 60%. Where competition is intense (*c* = 0.767) high *K* is clearly the fittest strategy regardless of its prevalence (Fig. 5C), and will be selected for. However, as high *K* becomes more prevalent, it also decreases the mean fitness of all individuals, but those with low *K* are more severely affected (Fig. 5C). Although high *K* individuals maximise their fitness when at low frequency (apparent negative frequency dependence), alleles for the strategy actually experience positive frequency dependence as their fitness relative to lower *K* individuals increases with increasing prevalence high *K* (Fig. 5C). Interestingly, the spread of high *K* has the overall effect of decreasing mean population fitness. Where all individuals in the population adopt high *K* individual fitness is actually lower than had all individuals maintained low *K* (Fig. 5C).

### Experiment 3: Effects of Fitness on Outcomes of Dominance Interactions

In our model, the parameter *η* (see Details in Models and Table 1) is the power of the relative nutritional states (fitness; *F*) of the *i*^*th*^ and *j*^*th*^ individuals to predict the outcome of a dominance interaction between these individuals. Given that we are largely concerned with species for which ability in dominance interactions is strongly correlated with nutritional state (see Lihoreau et al. [5]), in the above results *η* is assumed to be high (*η* = 25). We now explore the effects of the intensity of competition (*c*) in scenarios where there is greater stochasticity in the outcome of contests over food; *η* = 20 and *η* = 10.

**Fig 6:**
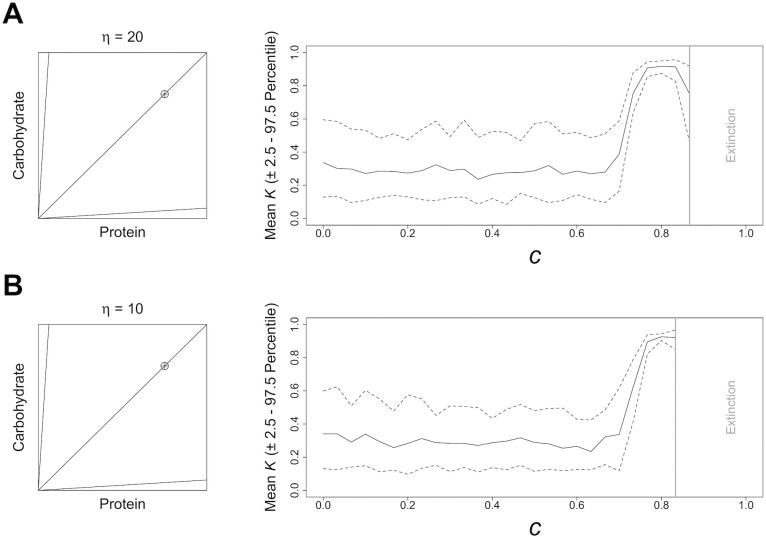
Model Results in Different 3-Food Environments with Reduced *η*. The effects of increasing competition, *c* (Equation 3) on the mean level of nutritional latitude, *K*, (and the 2.5^th^ and 97.5^th^ percentile; dashed line) that evolves under a 3-food environment, when the model is run with A) *η* = 20 and B) *η* = 10. All data are based on 30 model runs. Data from levels of competition, above which the population could not consistently survive (i.e., extinction, given by a bold grey line), have been removed. A geometric visualisation of the environment is given; see Fig. 2 legend for details.

Where *η* was set at a lower levels, increasing the intensity of competition had the same qualitative effect on the evolution of nutritional latitude (*K*) as described above; i.e. at low *c* a range of low levels of nutritional latitude appear optimal, but a transition to high *K* is favoured at *c* greater than 0.733 (Figs. 2 and 6). However, at lower levels of *η* the intensity of competition that lead to population extinction was decreased. With *η* = 10 the population could not consistently sustain itself above values of *c* of 0.833 (Fig. 6B). In an equivalent nutritional environment with *η* = 25 the population could not consistently sustain itself above values of *c* = 0.8667 (Fig. 2). These results indicate that having a stable dominance hierarchy, which is based on nutritional state can allow the population to better survive poor nutritional environments. We further discuss the biological implications of this finding below (see Future Directions in Discussion).

### Experiment 4: Truncated Selection

In the results described above, selection acts via two mechanisms. First, only those individuals able to attain fitness greater than 0.5 within 500 iterations are assumed to be in good enough condition to breed. Second, among those individuals fit enough to breed, fitness-proportionate selection operates [32]. The sensitivity of our results to this general selection mechanism was assessed by running the model with an alternative mechanism, truncated selection [29]. In this instance, the first 10% of the population to attain fitness over a cut-off are assigned as parents for the next generation. In our experiments cut-offs of 0.5 and 0.9 were assessed.

**Fig 7:**
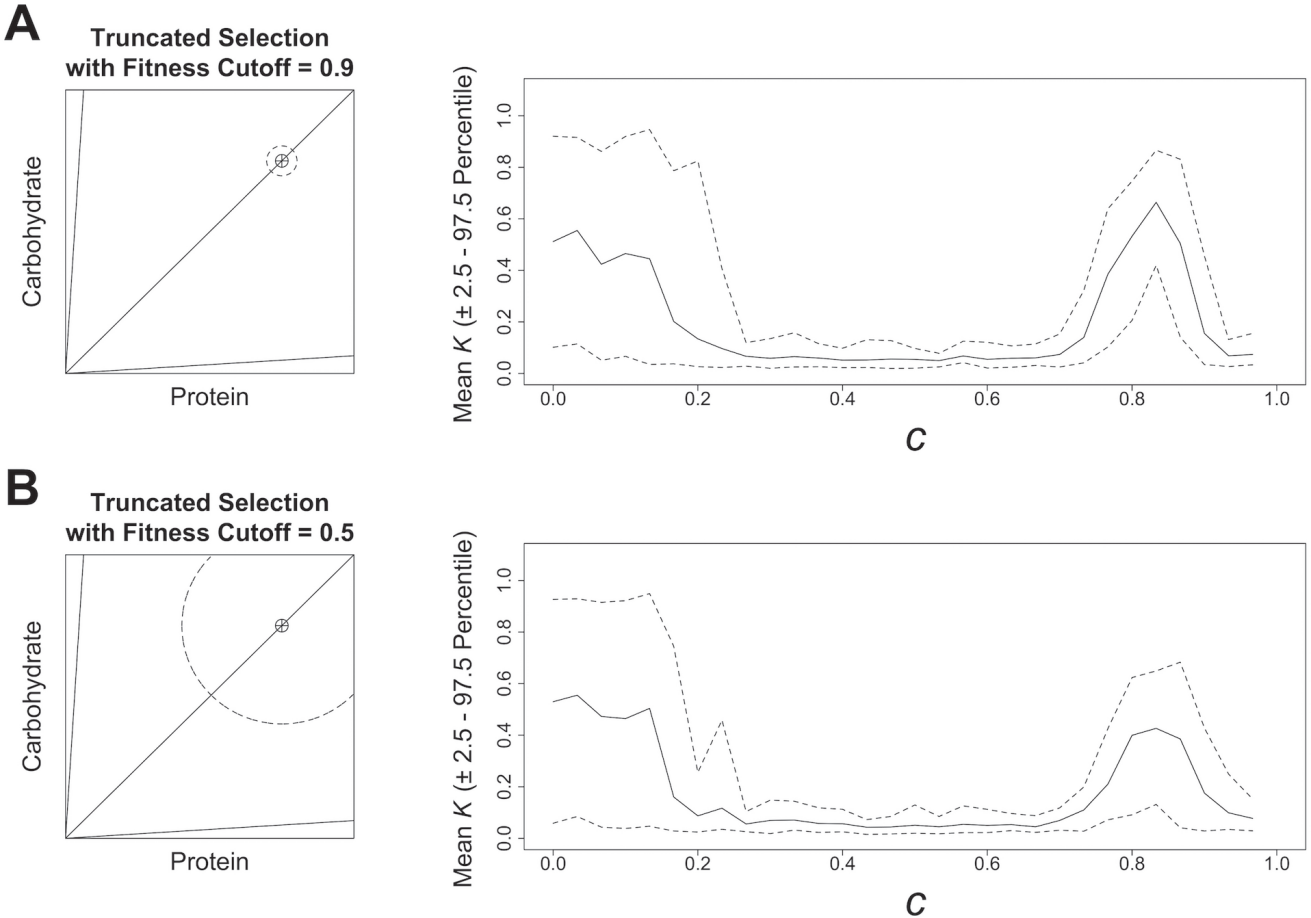
Model Results in Different 3-Food Environments with Truncated Selection. The effects of increasing competition, *c* (Equation 3) on the mean level of nutritional latitude, *K*, (and the 2.5^th^ and 97.5^th^ percentile; dashed line) that evolves under a 3-food environment, when the model is run with truncated selection. All data are based on 30 model runs. A geometric visualisation of each environment is given; see Fig. 2 legend for details. The first 10% of the population to cross the dashed line (*F* = 0.9 and 0.5; (A) and (B), respectively) in the geometric visualisation contribute to the subsequent generation.

Under truncated selection extinctions do not occur as the population is given a flexible amount of time to reach the fitness cut-off. We evaluated the effects of truncated selection on the model’s output in a 3-food environment with severely imbalanced foods (such an environment produced results typical of most other environments; Fig. 2B). In the absence of competition (*c* = 0) there was little or no selection on nutritional latitude: mean *K* = 0.5 with large variance (Fig. 7). However, low and moderate levels of competition selected for very low nutritional latitude (Fig. 7). As was the case under our general selection mechanism, a transition to increased nutritional latitude was still favoured under moderate to high competition (Fig. 7). However, *K* was not increased to anywhere near as high a level as under an equivalent nutritional environment with our general selection mechanism (Figs. 2 and 7). Finally, at very high levels of competition a return to low *K* was favoured (Fig. 7). The contrasting results of experiments 1 and 4 clearly illustrate that the mode of selection affects how nutritional strategies respond to contest competition. The implications of this finding for the biological interpretation of our model are discussed below (see Future Directions in Discussion).

## Discussion

### Model Outcomes and Biological Implications

We developed an ABM that combines principles of the GF with an evolutionary algorithm to explore how contest competition may affect the evolution of animal nutritional strategies. Specifically, we modelled the extent to which individuals consume nutritionally imbalanced foods that will not guide them directly to their intake target (nutritional latitude, *K*). In most of the nutritional environments we modelled, no competition and weak to moderate competition favoured low consumption of a suboptimal food. However, given that we observed high variance in stable values of *K*, it seems likely that there is no single optimal strategy. Rather, any fairly low level of nutritional latitude performs well. In contrast, moderate to severe competition appears to favour the consumption of more of an imbalanced food before seeking an alternative, than when competition is weak (i.e., they evolve increased nutritional latitude), potentially even consuming that food until reaching the point of nutritional compromise (see [23]). The balance of nutrients in the foods available also influences the optimal level of nutritional latitude. For example, in a 2-food environment that contained one highly imbalanced and one balanced food, a very low level of nutritional latitude was favoured regardless of competition (Fig. 3B). Thus, considering the nutritional composition as well as the amount of available foods is essential if we are to understand the role of competitive interactions in shaping the evolution of nutritional strategies.

Our model suggests that in social groups where the availability of nutrients is highly variable, plastic nutritional latitude should be adaptive so that individuals can alter their strategy in response to the intensity of competition. Several biological systems are well suited to empirical exploration of this idea. In social spiders, experimental evidence suggests that access to lipids governs reproductive asymmetry [13]. The manipulation described by Salomon et al. [13] (creating prey that vary in lipid content) could be employed, and then the behaviour of marked individuals within these groups observed (such as described in Whitehouse and Lubin [12]). An alternative model is the house cricket (*Acheta domesticus*), a species with well-studied nutritional requirements [33–38]. Males are known to compete with one another for food; moreover, sexual selection likely results in reproductive asymmetry, with larger males most likely able to meet the energy requirements for intra-sexual competition [39–43]. Contest-competition and aggression over food and mate access are also observable phenomena in male fruit flies (*Drosophila melanogaster*; [44,45]). This species also offers numerous other advantages including, being a model organism in genetics, being well studied with regards to its nutritional requirements and fitness consequences of nutritional imbalance and being suitable for artificial selection [1,2,46–48]. Using our ABM it will be possible to generate predictions for any number of nutritional scenarios specific to the model organisms described above. For example, considering spiders one may wish to explore a situation in which as food becomes scarce (i.e., competition increases in intensity), certain food rails appear only sporadically [49,50].

At the inter-species level our model suggests a role for contest competition and reproductive skew in shaping the evolution of dietary breadth. Specifically, consistent intense competition for access to a food containing a limiting nutrient, which results in reproductive skew, can select for high nutritional latitude, hence contributing to nutritional generalism. This hypothesis could be tested in a comparative nutrition framework (e.g., [31]), focussing on the intensity of contest competition and reproductive skew within groups of social generalists and specialists. Such approaches have, in the past, proved useful for studying the evolutionary mechanisms underlying dietary breadth, specifically suggesting that nutritional heterogeneity may lead to the adoption of specialist/generalist-specific rules of compromise ([23]; further discussed in Future Directions.).

Additional to insights into evolving nutritional strategies, our model supports predictions that contest competition over foods can lead to dominance hierarchies and reproductive asymmetries in social groups, because dominant individuals monopolise key nutrients for reproduction [5,8,13]. Contests for limited food can cause between-individual variance in reproductive output, regardless of the level of nutritional latitude or the nutritional environment. Given an apparent link between limited resources and alloparental care [51], contest competition over nutrients may be a mechanism forcing groups of animals on to the continuum from cooperative breeding, where helpers occasionally provide care to the offspring of breeders, to eusociality, characterised by a complete division of labour [52]. We note that our models represent a scenario in which individuals are unable to leave the group, even when competition becomes strong, due to some unstated ecological constraint. If future models explicitly focus on how nutrition and contest competition contribute to the evolution of sociality, they will likely want to vary the strength of constraints that keep individuals within the group.

Our models highlight some interesting relationships between nutrition, individual-level fitness and mean population fitness. Specifically, these models show that where individual nutritional state is a strong predictor of performance in dominance interactions (here *η*) and in turn reproductive asymmetry (i.e. a high variance in fitness), the population is better able to survive when nutrients are severely limiting. Accordingly, previous theoretical and experimental studies in social spiders have also suggested a strong dominance hierarchy ensures that the colony is able to survive resource poor periods, as at least a few females are able to monopolise enough nutrients to breed [8,11]. We also note that the spread of high nutritional latitude under strong competition bears some similarities to an evolutionary “tragedy of the commons” [53], because once the strategy becomes highly prevalent the mean fitness of the population becomes depressed to a lower level than might be the case if all (or the vast majority of) individuals to maintain low nutritional latitude.

### Future Directions

Our evolutionary model could be further expanded to give a more detailed representation of specific biological systems. First, we assumed that the fitness payoffs surrounding the IT are symmetrical. Geometric nutritional studies have shown that in some instances the fitness landscapes associated with the intake of nutrients may be asymmetrical [25]. A case in point is the predatory ground beetle (*Anchomenus doralis*), where a female’s egg production displays an asymmetrical response to protein and lipid intake when mapped as a response landscape onto a protein-lipid nutrient-space [54]. Models considering the effects that asymmetrical fitness landscapes have on the evolution of nutritional strategies themselves, and in turn the consequences for social structure, are particularly exciting avenues of investigation. Second, geometric nutritional studies also demonstrate that different species follow different rules of compromise (the extent to which they consume excesses of one nutrient relative to the IT to gain another which is limiting in the diet). The model described here conforms to what is known as the ‘nearest distance’ rule of compromise [23]: individuals seek to attain a nutritional state that minimises the Euclidean distance from the IT (see Models). Some species, such as the migratory locust (*Locusta migratoria*), appear to conform to such a rule of compromise when confined to a single food [31]. However, other rules of compromise are also followed. For example, the desert locust (*Schistocera gregaria*) follows what is known as an ‘equal distance’ rule of compromise, eating more of an imbalanced diet, over-consuming the excess nutrient to a greater degree (and under-consuming the deficient nutrient to a lesser degree) than *L*. *migratoria*, under the same no-choice experiment [31]. Evidence from this and other examples using the comparative approach suggests that the adoption of these two different rules of compromise closely associates with dietary breadth, with nutrient specialists adopting the nearest distance rule we implement here [23]. The study of the co-evolution of nutritional rules of compromise, dietary breadth, fitness-landscapes and other nutritional strategies (e.g. nutritional latitude) remains largely theoretical [23,25]. However, with the increasing application of nutritional geometry to a wider range of species, both in the lab and in the field, the comparative studies required to untangle the co-evolution between the aforementioned traits should soon be possible [23].

Within the selection mechanism implemented here, individuals must first attain a certain nutritional state to breed. Amongst those individuals with a high enough fitness to breed, relative fitness (determined by proximity to the IT) then governs overall representation in the subsequent generation (i.e., fitness proportionate selection; [32]). Thus, what we term the ‘general’ selection mechanism is most analogous to systems where reproductive asymmetries arise when resources become limiting. This mechanism of selection is typical of experimental outcomes in some social systems. For instance, female social spiders that do not attain enough nutrients (lipids) to reach a mature size at the end of the season are not capable of breeding, and larger individuals produce more offspring ([8,13,55–57] c.f. [15]). We also explored the effects of truncated selection on the model output. Whilst these two selection regimes produced some broadly similar results, there were also differences; namely, with truncated selection there was a lack of selection on nutritional latitude in the absence of competition, but low nutritional latitude was favoured under even weak competition. Truncated selection is most analogous to social systems, where dominance hierarchies and reproductive asymmetries are always present, regardless of food availability. For example, in eusocial wasps (*Polistes*; the inspiration for the original manifestation of the contest competition model we implement [27]) linear hierarchies form amongst females, with reproduction limited to the individual at the top (or the top few; [58]). For such species, where reproduction is always limited to a few individuals (perhaps those best able to track the IT), a model operating truncated selection may be most appropriate. Additionally, it occurs to us that artificial selection experiments can use truncated selection; i.e., the top few performing individuals are selected for breeding (e.g. [59]). In the future, geometric ABMs such as ours may be used to generate predictions for selection experiments on nutritional strategies. However, those models should explicitly incorporate truncated selection as other modes of selection may produce inaccurate predictions.

The models described here make simplifying assumptions about within population variation in nutritional requirements and the effects of nutritional state on fitness. For example, we only consider a single sex although sex differences in nutritional requirements may be ubiquitous (e.g. [60]). Such assumptions seem justifiable on the basis of the biological systems that we are interested in. Considering sex specifically, the relationship between contest competition, reproductive asymmetry and nutritional state is often only profound (or well understood) in one sex. For example, in populations of social spiders female sex ratio bias tends to be very strong and males seem largely absent [13,14]. Thus it seems reasonable to assume that males play a relatively minor role in competition over nutrient access. When modelling the relationship between nutritional state and other social phenomenon (e.g. collective behaviour and communal feeding [5]), however, it may be more realistic to model such variation. To incorporate this variation, rather than modelling a single intake target as we do here, one could include a bi-modal distribution of intake targets representing the differential requirements of each sex and individual heterogeneity simultaneously.

In this instance, the combination of evolutionary algorithms, ABMs and the GF has allowed us to produce testable experimental predictions for how intra-specific competition affects the evolution of nutritional latitude and dietary breadth. The wider application of this integrated approach could be applied to assess how other nutritional strategies and ecological factors interact [30]. For instance, Lihoreau et al. [5] explore how collective decision-making can optimise the nutritional decisions of entire groups, a phenomenon that may be applicable to animals exhibiting a range of social interactions [61–63]. By expanding that model with an evolutionary algorithm, it would be possible to generate predictions for how nutritional strategies and social phenomenon co-evolve. The next step in combining evolutionary algorithms with GF-based ABMs will be to use spatially explicit models [30]. In this way researchers will be able to model the evolution of nutritional strategies in complex environments that are closely representative of real world ecosystems.

### Models

Models were programmed in the software Netlogo [64]. Graphs were created and statistical calculations performed with *R* version 3.1.1 [65]. The model is described following the overview, design and details format of ABM description as widely recommended [66–68]. The code for the model can be found in S2 File.

#### Overview

##### Purpose

This model has been developed to explore how ecological factors affect nutritional strategies. Here, we have expanded the model of Lihoreau et al. [5] with an evolutionary algorithm to explore how intra-specific contest competition affects the evolution of nutritional latitude.

##### Entities, states, variables and scales

The model consists of individuals and their environment. As in the GF, the environment is a Cartesian plane (i.e., an *x*-*y* plane) representing the nutrient space. The nutrient space is made up of two nutrients, which are represented by (*x*, *y*) coordinates. We refer to the macronutrients protein (P) and carbohydrate (C), although our results are not specific to P and C, and can generally be thought of as any two nutrients. An individual’s (*x*, *y*) position in the nutrient space is its nutritional state. Individuals aim to navigate their nutritional state from an initial point of (0, 0) to the intake target (IT); an (*x*, *y*) coordinate that represents the ideal amount and balance of P and C (see examples in Fig. 1).

Individuals must eat foods (*f*) to move through the environment towards the IT, but can only feed on one food at a time. Thus, individuals must move in parallel with the food rail on which they are feeding (*α*_*f*_; Fig. 1; all parameters are described in Table 1). An individual may leave an inadequate food to find an alternative, the likelihood of which is governed by the parameter *K* (i.e., the nutritional latitude). An individual’s *K* is genetically determined by a single inherited mutable value. The overall amount of foods available may be limited. To eat a food that is ‘at capacity’, that is, which already has a full complement of consumers, an individual must first displace another *via* a dominance interaction. An individual’s ability in dominance interactions is governed by its fitness (*F*), which is a function of its distance to the IT (closer = fitter). By adjusting the abundance of food (*a*), we are able to manipulate the level of contest-competition (estimated as Equation 3 below). After 500 iterations a new generation begins, which is made up of offspring with *K* values inherited (with mutation) from the fittest individuals from the previous generation. The model runs for 1000 generations under varying levels of contest-competition, and we measure the evolution of nutritional latitude (*K*).

**Fig 8:**
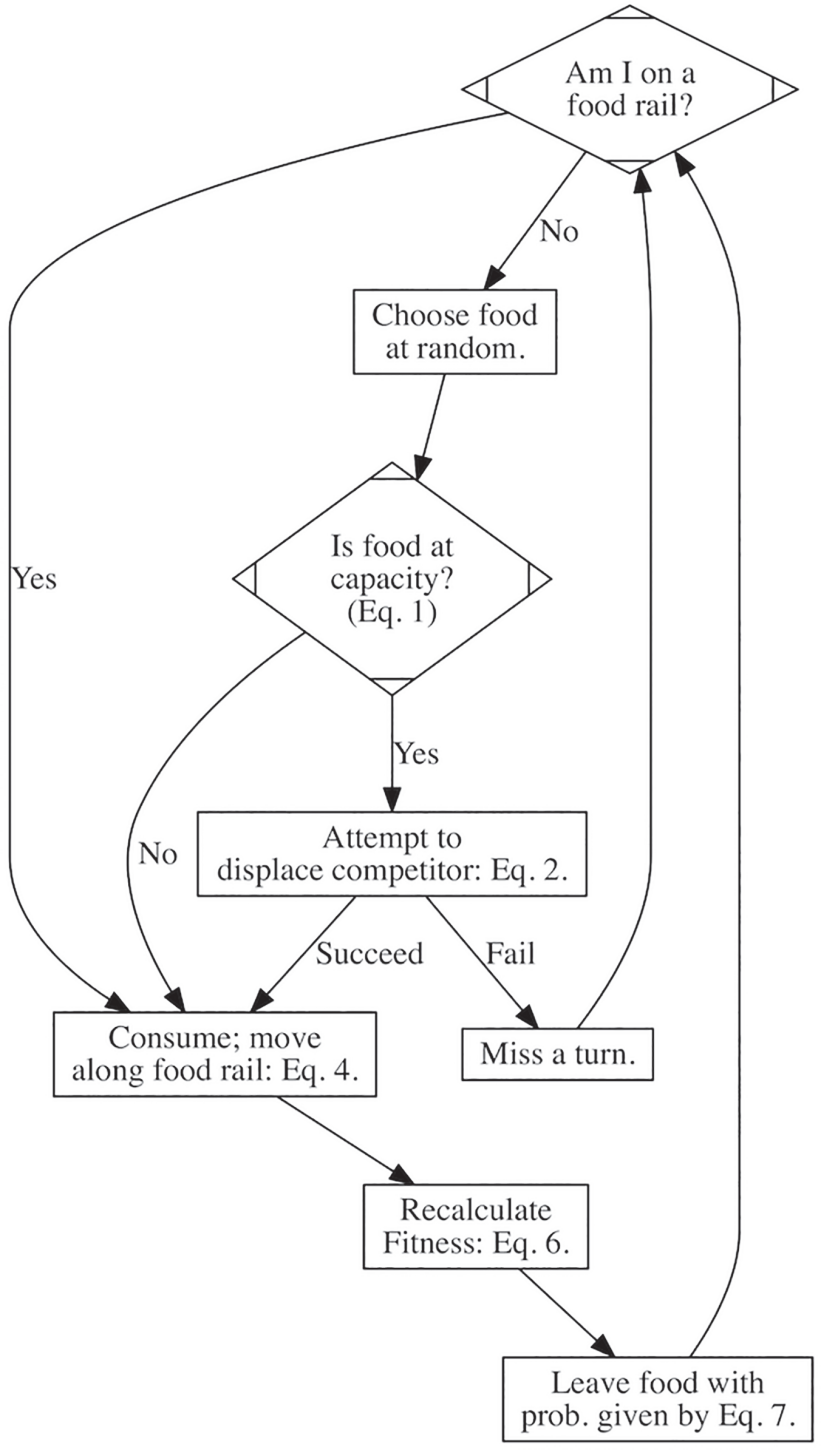
Flow Diagram of Model Processes. A flow diagram of the model. After 500 iterations of these processes, a new generation begins. Note that all individuals undergo each process before the model moves on to the next process (i.e. for the details of each process see Details in Models) and individuals are processed in a randomised sequence.

All model parameters described above and below are summarised in Table 1 and their mode of action is outlined in detail below (see Details below).

##### Process overview and scheduling

At every time step individuals perform the following four processes (name of process given in parentheses) in the given order: (1) those without a food source select a food at random and fight for it if necessary (‘Choose Food’), (2) calculate their appetite and eat (‘Eat’), (3) recalculate their fitness based on their nutritional state (‘Calculate Fitness’) and (4) may spontaneously leave a food source with a probability that is function of nutritional latitude, *K* (‘Leave’). An overview of the model is given in Fig. 8. Finally, after a 500 time steps a new generation is spawned from the previous generation, and the parental generation dies (‘Reproduce’). Individuals are processed in random order and each individual’s state variables are updated immediately after it completes a process. All individuals complete each process before the model progresses to the next process.

#### Design Concepts

Emergence: We are interested in the emergence of the optimum value of nutritional latitude (*K*) under varying levels of competition (*c*).

Observation: Values of *K* are observed at the end of 1000 generations.

Objectives: The objective of all individuals is to move through the nutritional space to attain a nutritional state as close to the IT as possible after 500 time-steps.

Sensing: Individuals are able to sense the number of other individuals currently feeding on a given food.

Interaction: Individuals may displace one another from a food source via a dominance interaction.

Stochasticity: Several events are determined by Bernoulli trials, and thus occur with given probabilities, independently of any others. For example, the probability that one individual displaces another from a food source is a function of the fitness of the two individuals. Such functions are outlined in detail below (see Details below).

#### Details

##### Initialisation

At initialisation the IT is set, as are parameters that govern the nutritional content of foods (i.e., *V* and *α*_*f*_), and *N*_*ind*_ individuals are created. All individuals are initialised with *K* = 1. Individuals are given a location (i.e., a nutritional state) of (*x*, *y*) = (0, 0). In the model of Lihoreau et al. [5] the position of the IT is set at a distance of 1 from the initial nutritional state and individuals can eat a maximum (*φ*) of $\sqrt{2}/500$ per time step. To aid with visualisation in Netlogo, the IT is set at a position in the environment of (500, 500). This difference between our model and that of Lihoreau et al. [5] is corrected by rescaling *φ*.

##### Model processes

Choose food: As in Lihoreau et al. [5], individuals that are not located on a food select one at random and then assess whether the number of individuals feeding on the chosen food is equal to the capacity. The capacity is given by Equation 1:


$$\text{capacity}=\frac{a}{N_{food}}N_{ind}$$ (1)

If the selected food is below capacity, the individual may take a place on the food rail, ready to feed (see ‘Eat’ below). However, if a food is at capacity the individual must displace a randomly selected ‘opponent’ via a dominance interaction. The probability of the *i*^*th*^ individual displacing the *j*^*th*^ is given by *Q*_*ij*_. This probability is the same as that in the contest competition model of Bonabeau et al. [27], although in that model ability in dominance was determined by the abstract value ‘force’. Following Lihoreau et al. [5], individuals’ ability in contests is dependent on nutritional state as given by Equation 2:

$$Q_{ij}=\frac{1}{1+e^{-\eta (F_{i}-F_{j})}},$$ (2)

where *F*_*i*_ and *F*_*j*_ are the respective fitness of the two individuals (see ‘Calculate Fitness’ below) and all other parameters are as in Table 1. If the challenger is successful in displacing the opponent, then the challenger will take a place on the food and the opponent will be displaced. If the challenger is unsuccessful it will remain displaced for a time step. The variable *η* maybe thought of as the power of nutritional state (*F*) to predict success in contests over food. For the most part we assume that this value is high as we are specifically interested in how nutritional strategy is affected by food availability in systems where nutritional state is a strong predictor reproductive dominance (e.g. social spiders [16]). However, we also explored how a reduced *η* affects the models behaviour.

To manipulate the intensity of competition within the model, we varied the capacity (Equation 1) of foods to support the population via manipulating the value of *a* (the abundance of food; Table 1). Where foods were more abundant competition was weaker. To aid interpretation, competition (*c*) is then given by Equation 3:


$$c=1-\frac{a}{N_{food}}.$$ (3)

Thus, when *a* is equal to *N*_*food*_, individuals can move freely between food rails, and *c* = 0; i.e., competition is absent. Note that following Equations 1 and 3, *c* is 1 minus the proportion of the population that each food rail can support on one time step.

**Fig 9:**
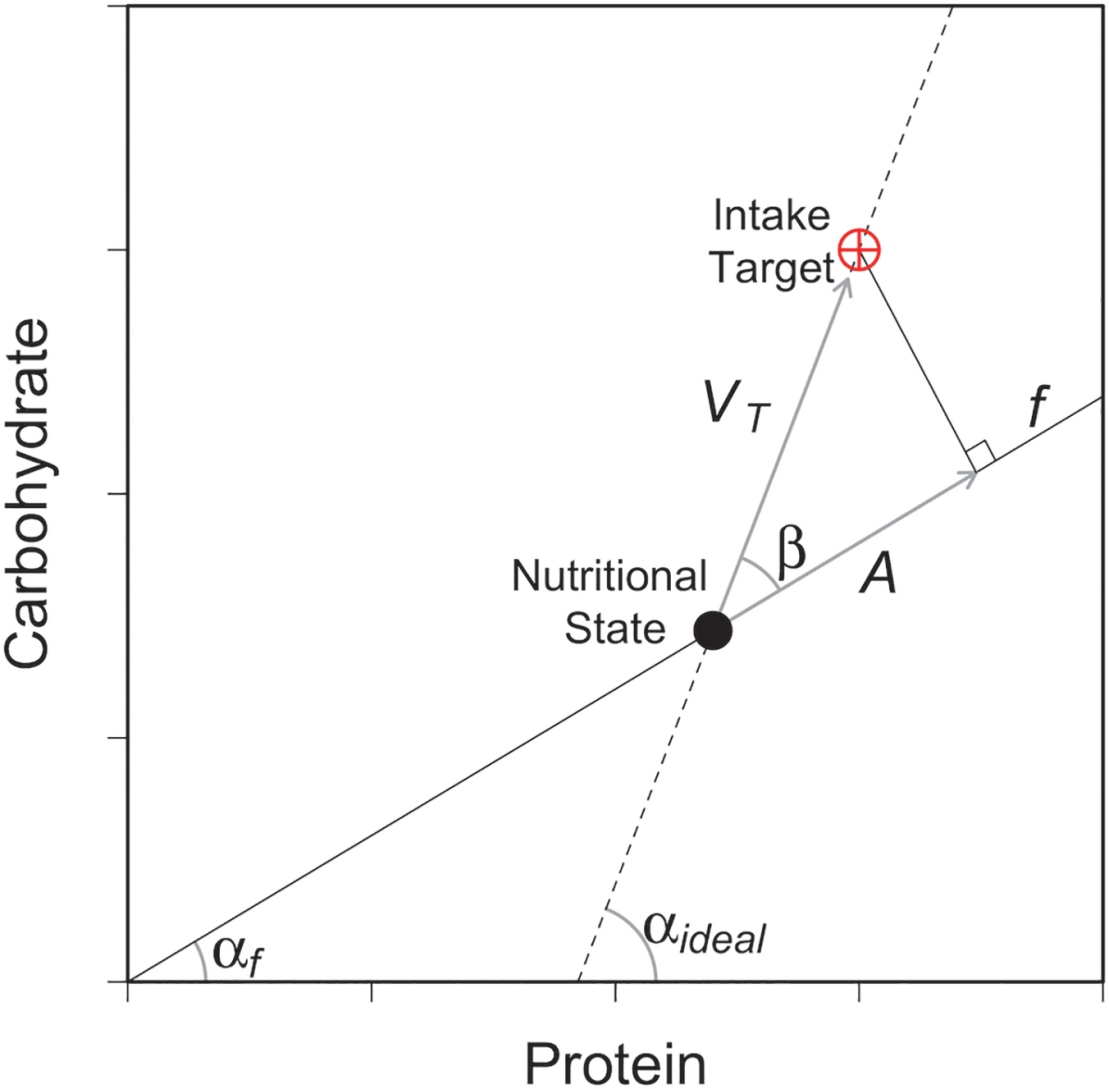
The Models Implementation of the Geometric Framework (GF). An example of our implementation of the GF, redrawn from Lihoreau et al. [5]. The *x* and *y* axes represent protein (P) and carbohydrate (C). The intake target (IT) is denoted by the red crosshair and the individual’s current nutritional state by the black point. The food rail for the food an individual is consuming is given by the black line (*f*) with the angle *α*_*f*_. The amount of food an individual would eat to maximise its fitness is given by the individual’s appetite (*A*). *A* is governed by the nearest distance rule of compromise; an individual gets as close to the IT as the food rail allows [21]. *A* is the scalar projection of the Euclidean distance between an individual’s nutritional state and the IT on to the food rail *f*. *A* is found by estimating the ‘ideal’ food rail that connects the individual’s nutritional state with the IT (dashed line with angle *α*_*ideal*_), the magnitude of the vector along which an individual would travel to reach the IT (||*V*_*T*_||) and the angle between *α*_*f*_ and *α*_*ideal*_ (*β*); Equation 4. Note that the amount an individual can eat in one time step has a maximum value of *φ*.

Eat: The ‘Eat’ process is summarised in Fig. 9. Eating consists of moving through the nutrient space at an angle (or heading) given by the food rail for the food on which the individual is eating; *α*_*f*_. The distance an individual moves (i.e., the amount eaten) is governed by the individual’s appetite (*A*), which is assumed to conform to the ‘nearest distance’ rule of compromise [23] and the maximum amount of food an individual can eat (*φ*) following Equation 4:

distance
moved=min{A,φ}, (4)

where *A* is calculated as Equation 5:

$$A=\left\|V_{t}\right\|\cos\beta ,$$ (5)

where *β* is the angle between the food rail on which the animal is feeding (*α*_*f*_) and the ‘ideal’ food rail that joins the individual’s current nutritional state with the IT (*α*_*ideal*_) and *V*_*T*_ is the vector connecting the individual’s current nutritional state and the IT; *A* thus gives the distance the individual would move to reach the point of nutritional compromise (Fig. 9). If this distance is greater than the maximum amount the individual can eat in one time-step (*φ*) then the individual’s nutritional state moves by *φ*.

Calculate fitness:The IT is the point in the nutrient space that maximises biological fitness. As in Lihoreau et al. [5] we assume that fitness (*F*) is a function of the distance between an individuals nutritional state and the IT, as given by Equation 6:


$$F=e^{-\mu D_{N}}.$$ (6)

Thus, as an individual nears the IT from any direction in the nutrient plane, its fitness increases towards a maximum value of 1. Here we correct *D*_*N*_ to be the proportion of the total distance an individual must move from the beginning of the simulation to reach the IT, thus making our model equivalent to that described in [5].

Leave: The ‘Leave’ function is the same as that implemented in Lihoreau et al. [5], although here *K* (nutritional latitude) is an individual-level variable rather than a fixed global parameter, and *K* as we model it here is equivalent to 1—*K* in Lihoreau et al. [5]. Individuals have a probability (*P*_*leave*_) of spontaneously leaving a food before having satisfied their appetite. This probability is governed by the quality of the food and *K*, following Equation 7:

$$P_{leave}=(1-K)\frac{\left|\alpha_{ideal}-\alpha_{f}\right|}{0.5\pi }+\max\{0,(K\frac{\varphi -A}{\varphi })\},$$ (7)

where α_*f*_ and α_*ideal*_ are measured in radians. Thus, with decreasing *K* and an increasing angular difference between α_*f*_ and α_*ideal*_, an individual becomes more likely to leave the food before having satisfied their appetite.

Reproduce: After 500 iterations of the above processes, a new generation begins. *N*_*ind*_ individuals are created and these offspring have their nutritional state set to (0, 0). Parents for the new generation are assigned via two mechanisms of selection. First, only those individuals that have been able to attain a fitness (*F*) value greater than 0.5 are assumed to be in good enough condition to breed; one may consider this equivalent to having attained enough nutrients to have developed to maturity (e.g. the final moult in female social spiders which appears to be a function of nutrition [8]). Second, among those individuals who have a fitness over 0.5, fitness proportional selection operates [29]; i.e., an individual with a higher fitness has a greater probability of being selected as a parent. Each offspring ‘selects’ a parent and inherits that individual’s level of *K*. This value of *K* then mutates, by adding a value drawn from a random normal distribution with a mean of 0 and a standard deviation of 0.025, and *K* is bounded between 0 and 1. *K* mutates as a random walk, thus, in the absence of selection, all values of *K* have an approximately uniform probability of arising. Note that under this selection regime it is possible that no individual is able to reach fitness of 0.5 within 500 iterations (e.g., where competition is severe). In such a situation, we consider the environment so nutritionally poor that the population is driven to extinction. Data from such replicates are disregarded, but these replicates are noted in the results.

The number of iterations (here 500) that make up a generation is equivalent to the number of feeding opportunities before the end of the reproductive period (e.g. the breeding season in social spiders [13]; see Introduction). The generation time is thus only meaningful when considered alongside the distance through the nutrient-space that an individual must cover to reach the IT, and the amount of food it is possible to eat at each feeding opportunity (i.e. here from an initial *x*, *y* (0, 0) to the IT (500, 500) given *φ* = 2). Here we use 500 iterations because in most biological circumstances, it seems realistic to assume that animals may reach the IT via an indirect route without a fitness penalty (for many species there is no one food with a perfect nutritional balance). Alternatively, it may not be necessary to eat at every available opportunity in order to reach the IT. Thus, 500 iterations allow individuals to reach their IT via a range of routes through the nutrient space, without necessarily compromising their fitness.

A more stringent approach would be to make the IT the furthest point along the ideal food rail that an individual could travel in one generation, assuming they eat at each iteration. Given that the IT may be viewed as the point in the nutrient space that maximises fitness one may, in some circumstances, justify the assumption that, ‘if individuals only ever ate foods with the ideal nutritional balance at each available opportunity for their entire lifetime they would maximise fitness’; i.e. reach the IT. Note that under this circumstance one is making the assumption that it is impossible to ‘over eat’ an ideal food. To test this more stringent set of assumptions we could alter either the position of the IT or the number of iterations in a generation. We repeated all of the above experiments with a shorter generation time (354 iterations) for a fixed IT and *φ*. These models produced the same qualitative conclusion; i.e. intense competition favours high nutritional latitude, although there were some quantitative differences (see S1 File).

The general selection mechanism that we implement is representative of the biological systems on which our models are based; i.e. systems where variability in reproductive success within a cohort can be attributed to access to nutrients. However, the sensitivity of our results to the ‘general’ selection mechanism was also further assessed by running the model with truncated selection [29]; see Future Directions in Discussion for a discussion of where the two approaches might be most appropriate.

#### Model Testing

The model described here is determined to produce similar output as that in Lihoreau et al. [5] by comparing the nutritional distribution of individuals with a single model run (comparison between S1 File and Fig. 5.A in Lihoreau et al. [5]). We then performed exploratory analyses of the model, which consisted of running the model over ten thousand generations, recording the mean and *K* at the end of each generation. These results suggest that within 1000 generations *K* reaches a stable equilibrium (S1 File). Nutritional environments and levels of competition (*c*) were manipulated by varying the parameters (*N*_*food*_, *V* and *a*).

## Supporting Information

S1 File Supplementary Results, Figs S1, S2, S3, S4, S5 and S6. (DOCX)

S2 File Netlogo model code and programming notes. (TXT)
