# Supplementary material for: Evolving Nutritional Strategies in the Presence of Competition: A Geometric Agent-Based Model
Source: PLoS Comput Biol. 2015 Mar 27;11(3):e1004111. doi: 10.1371/journal.pcbi.1004111 (PMC4376532; doi:10.1371/journal.pcbi.1004111)
Supplement: S1 File — (DOCX) [file pcbi.1004111.s001.docx]

# Evolving Nutritional Strategies in the Presence of Competition: A Geometric Agent-Based Model: Supporting Results

Alistair M. Senior^1,2,*^, Michael A. Charleston^3^, Mathieu Lihoreau^4^, Camille Buhl^1,2,5^, David Raubenheimer^1,2,6^ and Stephen J. Simpson^1,2^

1. The Charles Perkins Centre, The University of Sydney, Sydney, NSW, Australia.
2. School of Biological Sciences, The University of Sydney, Sydney, NSW, Australia.
3. School of Information Technologies, The University of Sydney, Sydney, NSW, Australia.
4. Centre National de la Recherche Scientifique (CNRS), Centre de Recherches sur la Cognition Animale, Université Paul Sabatier, Toulouse, France.
5. School of Agriculture, Food and Wine, The University of Adelaide, SA, Australia
6. Faculty of Veterinary Science, The University of Sydney, Sydney, NSW, Australia.

* Corresponding Author

email: alistair.senior1985@gmail.com (alt: alistair.senior@sydney.edu.au)

Tel: +61 2 8627 1616

Address: L4, Building D17, John Hopkins Drive, The University of Sydney, Camperdown, NSW, 2006.

## Supplementary Results

### S.1. Experiment 1: Nutritionally Complementary 2-Food Environments

In addition to the 2 and 3-food environments described for experiment 1 we also explored 2-food environments containing nutritionally imbalanced but complementary foods. In these environments, the response of *K* to increasing *c* resembled that in the 3-food environments: low but variable under weak/moderate competition, with a transition to high *K* at moderate to high *c* (Fig S.1). However, increasing the severity of the imbalance of the two complementary foods appeared to reduce the level of *c* that induced a transition to high *K*. To evaluate how nutritional imbalance affects the transition between *K* values, we systematically varied the level of imbalance in two complementary foods at values of *c* = 0.4 through 0.8. This analysis produced two insights. First, under weak to moderate competition, mean *K* increased when the environment contained increasingly more-imbalanced foods (Fig. S.2); hence at *c* = 0.4 with very mildly imbalanced food mean final *K* was 0.22 (Fig S.2A), but with highly imbalanced foods mean final *K* was 0.35 (Fig. S.2F). Second, the level of *c* that selected for a transition to high *K* was slightly lower when the environment contains severely imbalanced foods than when it contained only mildly imbalanced foods; with mildly imbalanced foods *K* increased at around *c* = 0.58 (Fig S.2A), but increased at *c* = 0.51 with highly imbalanced foods (Fig. S.2F).

### S.2. Mechanisms in 2-Food Environments Containing Balanced and Imbalanced Foods

To understand the mechanism underlying the results observed in Experiment 1 in an environment with a balanced and imbalanced food (Fig. 3), we allowed the model to run for one generation (500 iterations), with 150 individuals, half of which expressed high nutritional latitude (*K* = 0.9), and half low nutritional latitude (*K* = 0.1). The performance of these two strategies was assessed in the absence of competition (*c* = 0) and at a high level of competition (*c* = 0.8).

The distribution of the nutritional states of individuals employing each level of nutritional latitude in the nutrient space at the end of one generation is summarised in Fig S.3. Individuals with low nutritional latitude tended to maintain a nutritional state closer to the nutrient rail passing directly through their IT, while individuals with high nutritional latitude ‘wandered’ around the nutrient space. In the absence of competition, almost all individuals attain high enough fitness to breed, regardless of the environment or their level of nutritional latitude (Figs S.3A and S.3C, except for one individual in the latter). However, individuals with low nutritional latitude ended up, on average, closer to the IT (Figs S.3A and S.3C). As a result, in the absence of contest competition, low nutritional latitude is selected for.

When competition is strong, the nutritional environment becomes decisive in determining optimal nutritional latitude. An environment containing a food with a mild nutritional imbalance favours high nutritional latitude. Here, competition for access to the food with an optimal nutritional balance means that individuals with low nutritional latitude progress slowly through the nutrient space: only 20% of these individuals had a fitness high enough to breed in our experiment (Fig. S.3B). However, individuals with high nutritional latitude progress quickly through the nutrient space on the imbalanced food. As this food has only a mild imbalance, many individuals with high nutritional latitude attain fitness over 0.5 (40% in Fig. S.3B). In contrast, when the environment contains a food with a severe imbalance, individuals expressing high nutritional latitude become diverted away from the IT (Fig. S.3D). This diversion results as high *K* individuals consume much of the severely imbalanced food, and with no complementary alternative available, the resulting imbalanced nutritional state cannot be rectified. As a consequence, in the experiment we ran, 96% of the individuals with high nutritional latitude did not attain a level of fitness high enough to breed when the environment contained severely imbalanced food (Fig. S.3D).

## S.3. Experiments with a Shorter Generation Time

To test the models sensitivity to the generation time (500 iterations in the main text), we re-ran all experiments with a shorter generation time. Here we use 354 iterations, which makes the IT the furthest point along the food rail for the food with an ideal nutritional balance an individual can travel before the end of the simulation (the biological significance of this generation time is described in section 4.3.). Fig. S.4 gives the results for the repeat of experiment 1 in 3-food environments (comparable to Fig. 2 in the main text). As shown, with a shorter generation time the results remain qualitatively similar to those with a generation time of 500 iterations; where *c* is set at low to moderate values low values of nutritional latitude (*K*) are selected for, but at higher values of *c* a transition to high *K* occurs (Fig. S.4). An observable effect of decreasing the generation time is that the levels of competition above which the population is unable to sustain itself is lower with a decreased generation time than with a 500 iteration generation time (for a comparable nutritional environment; Figs 2 and S.4). This finding is not necessarily surprising as a longer generation time, there is a greater likelihood that individuals will attain a nutritional state that allows them to reach maturity (here *F* > 0.5), before the end of the breeding season.

## Figure S.1

**Model Results in Different 2-Food Environments:** The effects of increasing competition, *c* (Equation 3) on the mean level of nutritional latitude, *K*, (also showing the 2.5^th^ and 97.5^th^ percentile; dashed line) that is stable under nutritional environments differing nutritional environments containing 2 foods. All data are based on 30 model runs. Data from levels of competition, above which the population could not consistently survive (i.e. extinction, given by a bold grey line), have been removed. A geometric visualisation of each nutritional environment is also depicted, where lines and a crosshair depict food rails and the intake target, respectively.

## Figure S.2

**Model Results in Systematically Varied 2-Food Environments:** The effects of increasing competition, *c* (Equation 3), between levels of 0.4 and 0.8 on the mean level of nutritional latitude, K, (and the 2.5th and 97.5th percentile; dashed line) that is stable under a nutritional environment with two foods that increase in the severity of their nutritional imbalance, but are complementary. A geometric visualisation of each environment is given in each panel; see Fig. S.1 legend for details.

## Figure S.3

**Nutritional States of Individuals at the End of One Generation:** The distribution of individuals within the nutrient space after 500 iterations of the model (i.e., one generation). Each population consisted of 150 individuals, 75 with *K* = 0.9 (red) and 75 with *K* = 0.1 (black). The intake target is depicted by a crosshair and the foods available by food rails. The minimum fitness requirement for breeding (fitness > 0.5) is depicted by a dashed circle. Models were run with both no competition (*c* = 0; (A) and (C)) and with strong competition (*c* = 0.8; (B) and (D)).

## Figure S.4

**Model Results in Different 3-Food Environments with Generation Time of 354 Iterations:** The effects of increasing competition, *c* (Equation 3) on the mean level of nutritional latitude, *K*, (also showing the 2.5^th^ and 97.5^th^ percentile; dashed line) that is stable under nutritional environments differing nutritional environments containing 2 foods. All data are based on 30 model runs. Data from levels of competition, above which the population could not consistently survive (i.e. extinction, given by a bold grey line), have been removed. A geometric visualisation of each nutritional environment is also depicted, where lines and a crosshair depict food rails and the intake target, respectively.

## Figure S.5

**The Nutritional State of Individuals Under Differing Levels of Competition:** The distribution of 300 individuals within the nutrient space when the first individual reaches fitness greater than 0.975. Amount of protein (P) is shown on the *x*-axis and amount of carbohydrates (C) is shown on the *y*-axis. Three food rails (P:C of 1:*V*, where *V* = 0.625, *V* = 1 and *V* = 16) are represented by solid black lines. Different combinations of characters and colours represent different levels of food abundance (*a*). Higher *a* indicates an environment with a higher abundance of food, and thus lower competition. Comparisons between the nutritional distribution of individuals represented depicted and those in Figure 5.A of Lihoreau et al. [5] suggests that the two models produce similar output. Note that differences in scaling between the *x* and *y* axes in the two figures are due to a small programming difference that is accounted for (see ‘Initialisation’ in the main text), and that what we refer to as *a* is termed *c* in Lihoreau et al. [5]. All other parameters are equivalent to Lihoreau et al. [5].

## Figure S.6

**The Evolution of Nutritional Latitude (*K*) Over One Thousand Generations:** A trace of the evolution of *K* with competition (*c* = 0.5) and a 3-food nutritional environment (*V* = 0.625, *V* = 1 and *V* = 16), a GF depiction of which is also shown. *K* is the mean population level at the end of a generation, with a population size of 150. Three model runs are shown, where individuals were initialised with *K* = 1 (black line), *K* = 0.5 (red line) and *K* = 0 (green line). Within 1000 generations all model runs converge on similar evolved *K*.
